# GLI inhibitor GANT-61 diminishes embryonal and alveolar rhabdomyosarcoma growth by inhibiting Shh/AKT-mTOR axis

**DOI:** 10.18632/oncotarget.2569

**Published:** 2014-10-31

**Authors:** Ritesh K. Srivastava, Samer Zaid Kaylani, Nayf Edrees, Changzhao Li, Sarang S. Talwelkar, Jianmin Xu, Komaraiah Palle, Joseph G. Pressey, Mohammad Athar

**Affiliations:** ^1^ Department of Dermatology, University of Alabama at Birmingham, Birmingham, Alabama, 35294-0019, USA; ^2^ Division of Hematology/Oncology, Department of Pediatrics, University of Alabama at Birmingham, Birmingham, Alabama, 35294-0019, USA; ^3^ Department of Oncologic Sciences, Mitchell Cancer Institute, University of South Alabama, Mobile, Alabama 36604

**Keywords:** EMT, GLI1/2, GANT-61, m-TOR, Rhabdomyosarcoma, Shh signaling

## Abstract

Rhabdomyosarcoma (RMS) typically arises from skeletal muscle. Currently, RMS in patients with recurrent and metastatic disease have no successful treatment. The molecular pathogenesis of RMS varies based on cancer sub-types. Some embryonal RMS but not other sub-types are driven by sonic hedgehog (Shh) signaling pathway. However, Shh pathway inhibitors particularly smoothened inhibitors are not highly effective in animals. Here, we show that Shh pathway effectors GLI1 and/or GLI2 are over-expressed in the majority of RMS cells and that GANT-61, a specific GLI1/2 inhibitor dampens the proliferation of both embryonal and alveolar RMS cells-derived xenograft tumors thereby blocking their growth. As compared to vehicle-treated control, about 50% tumor growth inhibition occurs in mice receiving GANT-61 treatment. The proliferation inhibition was associated with slowing of cell cycle progression which was mediated by the reduced expression of cyclins D1/2/3 & E and the concomitant induction of p21. GANT-61 not only reduced expression of GLI1/2 in these RMS but also significantly diminished AKT/mTOR signaling. The therapeutic action of GANT-61 was significantly augmented when combined with chemotherapeutic agents employed for RMS therapy such as temsirolimus or vincristine. Finally, reduced expression of proteins driving epithelial mesenchymal transition (EMT) characterized the residual tumors.

## INTRODUCTION

Rhabdomyosarcoma (RMS) is the most common soft-tissue sarcoma of childhood [1, 2]. Based on distinct histological features, RMS are categorized into two major subtypes: embryonal (e)RMS and alveolar (a)RMS [3]. The incidence of eRMS (approximately 75% of the total cases) is highest in children aged younger than 5 years, whereas incidence of aRMS (approximately 16% of cases) does not vary significantly with age [4, 5]. The survival rates for aRMS and eRMS patients with metastatic and/or advanced disease are remarkably low [3]. Despite possibly a common mesenchymal origin, the differences in genetic, morphologic and therapeutic responses indicate that each RMS sub-type is a distinct entity [6]. aRMS is associated with recurrent chromosomal translocations. The translocation t(2;13)(q35;q14) leading to the PAX3-FOXO1 (P3F) gene fusion occurs in 55% of aRMS, the translocation t(1;13)(q36;q14) leading to the PAX7-FOXO1 gene fusion are present in 22% of cases, and remaining 23% are fusion-negative [5]. eRMS has not been associated with a recurrent diagnostic genetic alteration, although loss of heterozygosity at 11p15 is known to occur [7, 8]. However, sonic hedgehog (Shh) signaling is considered to be an important driver of about half of eRMS [9], particularly, the eRMS that develop in patients with Basal Cell Nevus Syndrome or Gorlin Syndrome [10].

Current therapeutic approaches have not resulted in significant improvement in outcome for patients with recurrent and/or metastatic disease. These patients still face a grim prognosis and better treatment approaches are desperately needed [11-13]. Existing treatment for RMS includes multi-agent chemotherapy, radiation therapy and surgical resection [14]. Introduced decades ago, the combination of vincristine, dactinomycin and cyclophosphamide (VAC) is still generally considered the standard of care for RMS [15]. For patients with very high risk disease, in particular those with metastasis at three or more sites, the outcome remain dismal with less than 20% of patients surviving [3, 16]. Intensifying chemotherapy in these patients has also not resulted in an improved outcome [17]. Thus, studies exploring the role of molecular target-based inhibitors may find more effective therapies, either as single agents or when combined with conventional chemotherapy backbones. Recent advances in the understanding of molecular alterations occurring in refractory and metastatic RMS provide opportunities to test novel therapeutic options [18-20]. In this regards, dysregulation in RAS pathway, insulin-like growth factor (IGF), hedgehog (Hh), p53 and AKT/mTOR signaling which have been shown to be associated with the pathobiology of RMS [21-26], may be categorized as novel targets. Earlier, we showed that augmenting p53-dependent apoptosis regulatory signaling may be effective in killing rhabdoid cells [21].

The Hh signaling, a fundamental signal transduction pathway comprises the interaction of the ligands Hh such as Sonic hedgehog (Shh), Desert hedgehog (Dhh) or Indian hedgehog (Ihh) with the receptor protein Patched (Ptch), that leads to the repression of another membrane-associated protein smoothened (SMO). This results in the activation of downstream effector transcription factors known as GLIs. Among them, GLI1 and GLI2 are activator and GLI3 is a repressor of the pathway [27]. In addition, under non-canonical settings, sustained activation of GLI1/2 is known to occur leading to oncogenic consequences involving dysregulation of cell cycle progression and proliferation [28]. Targeting these effector molecules may have some cancer modulating effects not only in RMS but also in other neoplasm [29-32].

In this study, we found that GLI1/2 expression is high in cells derived from both RMS sub-types, which prompted us to test whether these transcription factors are involved in disease progression of both alveolar and embryonal sub-types and blocking these targets may attenuate the neoplastic growth. The published data remain insufficient to address this question [23]. Our data show that targeted inhibitions of GLI1/2 by their known inhibitor GANT-61 results in the effective blockade of proliferation and promotes death of RD and RH30 cells derived from the two human RMS sub-types. The growth reduction in the aRMS and eRMS xenograft tumors by GANT-61 was similar. GANT-61 treatment reduced the progression of epithelial-mesenchymal transition. Mechanism by which GANT-61 affects EMT progression involved reduction of the effector proteins in both Shh and AKT/m-TOR signaling pathways. We also found similar results in xenograft tumors developed by poorly differentiated rhabdoid A2O4 cells which also manifested increased expression of GLI1. Rhabdoid tumors are highly aggressive early childhood neoplasm with limited therapeutic options. Additional novel observation in this study is the demonstration that the effects of GANT-61 are significantly augmented by the combinatorial treatment with mTOR inhibitors temsirolimus (Torisel) or rapamycin (RAPA) or mitotic inhibitor vincristine (VCR).

## RESULTS

### GANT-61 treatment inhibits human RMS tumor growth

**Figure 1:**
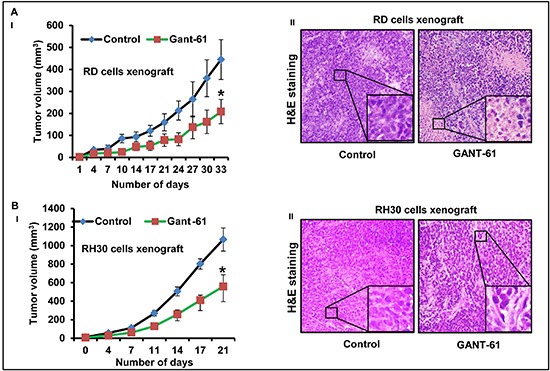
GANT-61 treatment inhibits eRMS (RD) and aRMS (RH30) cells-derived xenograft tumor growth **(A-I & B-I)** Line graph showing inhibitory effects of GANT-61 on the tumor volume of RD (A-I) and RH30 (B-I) cells-derived xenograft tumors. **(A-II & B-II)** H&E staining of the 5 μm sections of RD (A-II) and RH30 (B-II) xenograft tumors. Athymic nu/nu mice bearing RD or RH30 cells-derived xenograft tumors were treated with i.p injections of GANT-61 (50mg/kg, body weight in 200μl PBS; three times a week) whereas control mice bearing these tumors received an i.p. injection of vehicle. Photographs were captured at 40X magnification using Olympus BX51 microscope with Olympus DP71 digital camera. Insets represent magnified area of the images.

Both RD and RH30 cells following inoculation in nude mice form stable xenograft tumors. Animals receiving each cell-type were divided into two groups (n = 5, each group). Control mice group received an i.p. injection of vehicle, whereas experimental group of mice received i.p injections of GANT-61 (50mg/kg, body weight in 200μl PBS; three times a week). Dose selection for GANT-61 was made according to a previously published study [33]. Treatment of nude mice bearing RMS with GANT-61 reduced tumor growth significantly. The differences in the growth of tumors in vehicle- and GANT-61-treated groups were apparent beginning from the week second of tumor cell inoculation. At later time-points these differences in the tumor growth were more prominent and significant (*p < 0.05). GANT-61 inhibits the growth of both of these tumor sub-types with almost same efficiency. At the termination of the experiment, inhibition was about 53% in RD cells derived tumors (Fig. 1AI) and 47% in RH30 cells tumors (Fig. 1BI). The changes in tumor cells morphology following GANT-61 treatment was studied using hematoxylin and eosin (H&E). The histology of these tumors is shown in Fig. 1A-II and 1B-II. As compared to vehicle-treated controls, GANT-61-treated residual RD cell xenograft tumors showed prominent necrosis while, RH30 cells-derived tumors were more differentiated.

### GANT-61 treatment inhibits proliferation and induced apoptosis in human RMS xenograft tumors

**Figure 2:**
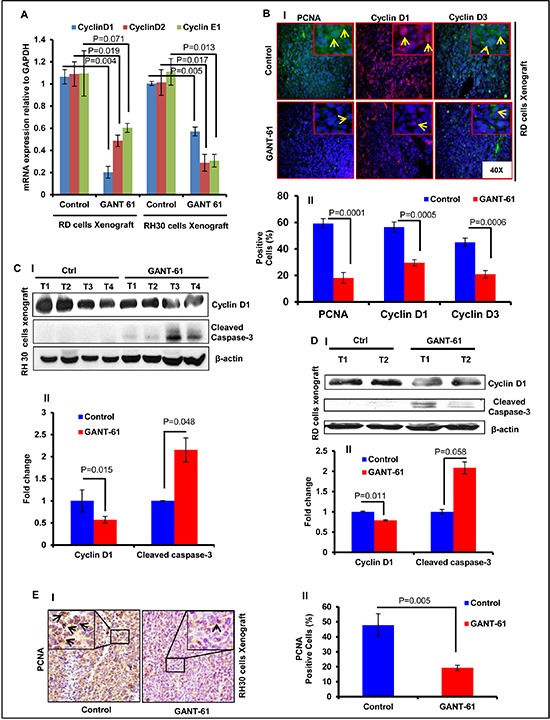
GANT-61 treatment reduces proliferation and induces apoptosis in RMS xenograft tumors **(A)** Real time PCR analysis of cyclin D1, D2 and E1 in GANT-61-treated xenograft tumor (RD) vs. vehicle-treated control tumors. **(B)** Immunofluorescence staining of PCNA, cyclin D1 and D3 in these xenograft tumors. Arrows indicate the positively stained cells. (C & D) Western blot analysis of cyclin D1 and cleaved caspase-3 in RH30 **(C)** and RD **(D)** cells-derived xenograft tumors. **(E-I)** Immunohistochemical staining of PCNA in vehicle- and GANT-61-treated RH30 xenograft tumors. **(E-II)** Histograms representing percentage of PCNA positive cells. *P* value represents the level of significant difference between GANT-61-treated and vehicle-treated controls. T1 to T4 represent tumors excised from 4 different mice. Histograms representing the densitometric analysis of western blot bands show significant differences in the protein expression when compared to vehicle-treated controls. Photographs were captured at 40X magnification using Olympus BX51 microscope with Olympus DP71 digital camera. Insets represent magnified area of the images.

First, we determined the biomarkers depicting proliferation and apoptosis in these tumors to investigate whether GANT-61 promotes inhibition of proliferation or/and induces apoptosis. Real time PCR analysis showed significant reduction in the expression levels of mRNA of proliferation-related cyclin D1/2 and E1 (Fig 2A). These data were supported by the immunofluorescence staining of RD cells-derived xenograft tumors section (Fig. 2B). GANT-61-treatment to mice bearing RMS xenograft significantly reduced the percentage of cells positive for PCNA (*p* = 0.0001), cyclinD1 (*p* = 0.0005) and cyclinE1 (*p* = 0.0006) staining as compared to vehicle-treated tumors (Fig. 2B-I) as also expressed as % positive cells in the histograms (Fig. 2B-II). Western blot analysis also showed similar results (Fig. 2C & 2D). Densitometric analysis of band intensity expressed as fold change showed significant differences in the expression of these proteins when compared to vehicle-treated controls (Fig. 2C-II & 2D-II). Immunohistochemical analysis showed that unlike vehicle-treated RH30 xenograft tumors, which have an intense wide-spread nuclear staining of PCNA, only a few cells were positive for PCNA in GANT-61 treated group (Fig. 2E-I & II). GANT-61 treatment augmented apoptosis in both of these tumor-types as inferred from the presence of multiple TUNEL-positive cells in the residual tumors from GANT-61-treated animals (data not shown). Consistently, enhanced cleaved caspase-3 expression was detected in the WB analysis of tissue lysates from both RD and RH30 cells-derived tumors (Fig. 2C & 2D). These data suggest that GANT-61 acts by blocking proliferation and by inducing apoptosis.

### GANT-61 inhibits cell cycle proteins, reduces colony formation and induces apoptosis in RMS cells *in vitro*

**Figure 3:**
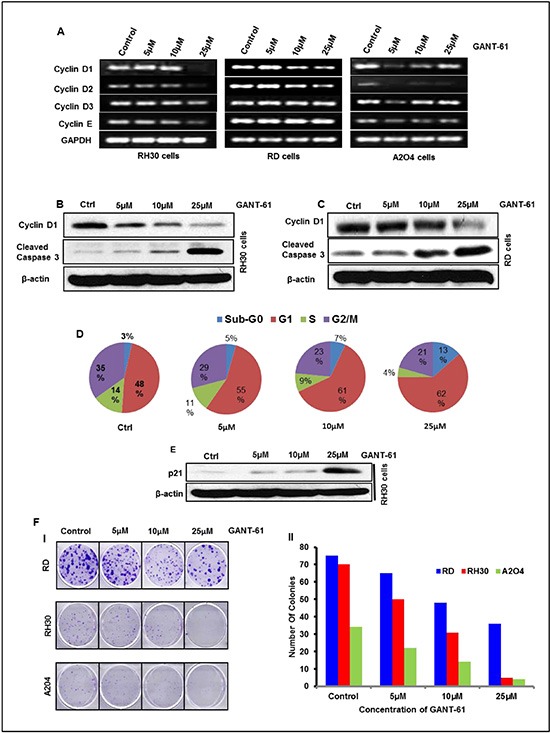
GANT-61 treatment to RMS cells inhibits proliferation, colony formation and induces cell cycle arrest/apoptosis in a dose-dependent manner **(A)** Reverse transcriptase PCR analysis of cyclin D1, D2, D3 and E genes. **(B & C)** Western blot analysis of cyclin D1 and cleaved caspase-3. **(D)** Pie chart showing effects of GANT-61 on RH30 cell cycle progression. Sub-G0 population represents dead cells. **(E)** Western blot analysis of cell cycle inhibitory protein, P^21^. **(F-I)** Effects of GANT-61 on clonogenic/colony formation assay of RD, RH30 and A204 cells. (F-II) Histograms depicting number of colonies developed following GANT-61 or vehicle treatments. Human RMS (RD & RH30) and rhabdoid (A2O4) cells were treated with various concentrations (5-25μM) of GANT-61 for 24 h and subjected to mRNA and protein analysis. Data are represented as % of cells present in different phases of cell cycle.

To confirm the *in vivo* results and to provide a firm basis to the mechanistic insight, we explored the effects of GANT-61 on cell cycle progression, colony formation and apoptosis in *in vitro* assays using these RMS cells in culture. MTT assay using various concentrations (0.5–250 μM) of GANT-61 was conducted to determine suitable concentration range of GANT-61 for further studies. Based on these results, we selected a concentration range of 5 to 25 μM to investigate its anti-proliferative and pro-apoptosis effects. GANT-61 treatment to RMS cells exhibited anti-proliferative effects and induced cell death in a dose-dependent manner (Supplementary Fig. S1A). GANT-61-treated cells were morphologically distinct from vehicle-treated cells. The morphological alterations in these cells included cell rounding, loss of cell adhesion, contraction of cytoplasmic membrane and blebbing (Supplementary Fig. S1B). Reverse transcriptase PCR analysis showed that treatment of RMS cells in culture with GANT-61 reduced expression of cyclins D1/2/3 and E. In addition to the reduction in the transcript levels of these genes, a similar decrease in the protein level of cyclin D1 was also observed both in GANT-61-treated RD and RH30 cells (Fig 3B & 3C). We also performed flow cytometry analysis to complement the observed effects of GANT-61 on cell cycle progression. GANT-61 treatment arrested these cells mainly in G0/G1 phase (Fig. 3D). With increasing concentrations GANT-61, significant increases in the proportion of cells in G0/G1 phase were recorded. Similar concentration-dependent effects were noticed in sub-G0 population. Moreover, lower concentrations of GANT-61 also manifested similar increases in dead cells but at later time-points of 48 and 72 h of GANT-61 treatment (data not shown). Consistent with the enhancement in sub-G0 populations, increase in cleaved caspase-3 in GANT-61-treated RD and RH30 cells occurred dose-dependently (Fig 3B & 3C). The G0/G1 arrest was correlated with the appearance of cell cycle inhibitory protein, p^21^ which is also known as CDK-interacting protein 1 (CIP1). It was found to be induced dose-dependently following GANT-61 treatment (Fig. 3E). This was further confirmed in colony formation/clonogenic assay, where GANT-61 reduced the number of colonies in a dose-dependent manner (Fig 3F-I & II).

### GANT-61-treated tumors show reduced expression of Shh and mTOR signaling related proteins

**Figure 4:**
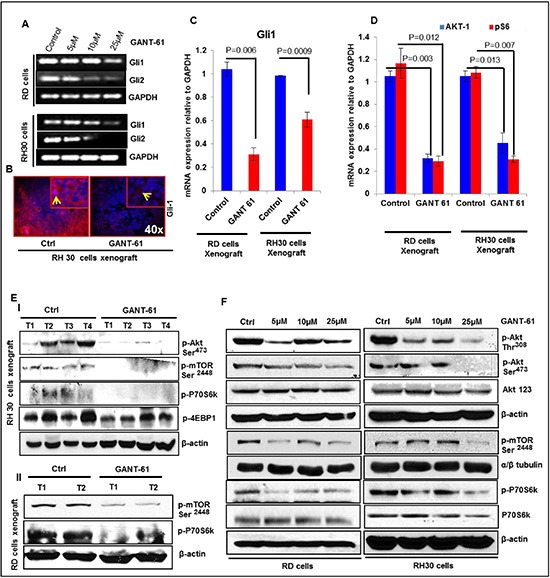
GANT-61 treatment dampens Shh and m-TOR signaling pathways **(A)** Semi-quantitative RT-PCR analysis of Gli1/2 in RD and RH30 cells at various concentrations of GANT-61. **(B)** Immunofluorescence staining of GLI1 in vehicle-and GANT-61-treated RH30 cells-derived xenograft tumors. **(C)** Real time PCR analysis of Gli1 mRNA expression relative to GAPDH as an internal control in GANT-61-treated xenograft tumors as compared to vehicle-treated control tumors. AKT/mTOR signaling pathway proteins in GANT-61-treated RD and RH30 cells-derived tumors vs. vehicle-treated control tumors: **(D)** Real time PCR analysis and **(E-I & EII)** western blot analysis. **(F)** Western blot analysis of GANT-61 on the phosphorylation of AKT/mTOR signaling pathway proteins (p-AKT, p-mTOR, p-P70S6K) in RD and RH30 RMS cells. T1 to T4 represents tumors excised from four different mice. *P* value represents level of significant difference in GANT-61-treated and vehicle-treated controls.

The pathogenesis of RMS is known to involve Shh and Akt/mTOR signaling pathways [22, 25, 34]. However, it remains unclear whether inhibitors of these targets effectively diminish this neoplasm. Here, first we analyzed the expression of Shh pathway genes in both eRMS (RD, SMS-CTR) and aRMS (RH30, CW0919) cells in addition to poorly differentiated A204 rhabdoid cells in comparison to normal human smooth muscle (HA-VSMC) cells (Supplementary Fig. S2). Based on real time PCR data, we inferred that as compared to HA-VSMC cells the expression of Gli1 and Ptch-1 was elevated at least by 2 fold in cells representing both RMS sub-types. Based on the relative expression of Gli1/2, Ptch-1/2 and Akt-1, we selected RH30 and RD cells which represent respectively aRMS and eRMS for further in-depth studies. It is known that the activation of GLIs occurs through canonical as well as non-canonical Shh signaling pathways [35]. In addition, crosstalk between Shh and Akt/mTOR pathway has also been described [18]. Here, we found that GANT-61 treatment not only decreased the expression of Glis but also diminished the expression of proteins in the mTOR signaling pathway as ascertained at by mRNA expression, western blot and immunofluorescence staining both *in vitro* in cells in culture and *in vivo* in xenograft tumors (Fig 4 and Supplementary Fig. S3). Interestingly, reduced phosphorylation of AKT at both Thr^308^ and Ser^473^ in GANT-61-treated RD and RH30 cells was observed while no significant change in the total AKT levels was noticed (Fig. 4F). These changes in GANT-61-treated RMS cells accompanied a decrease in cyclin D1 (Fig. 2).

### GANT-61 treatment inhibits epithelial mesenchymal transition (EMT)

**Figure 5:**
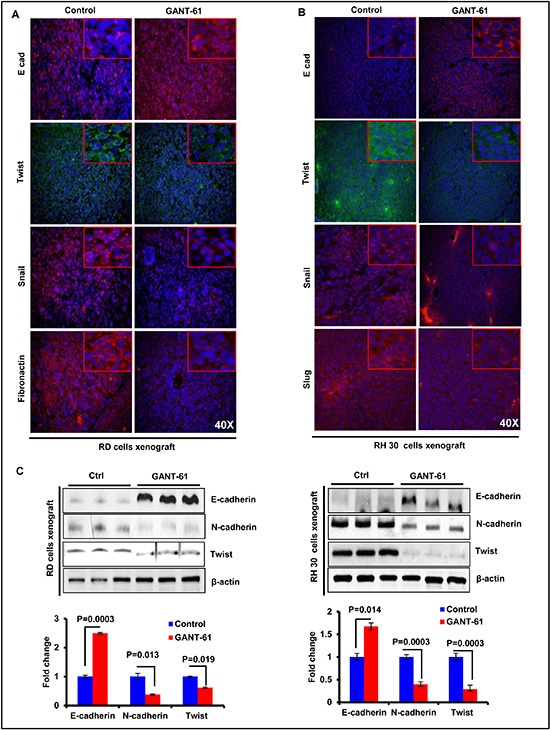
GANT-61 inhibits the expression of biomarkers representing epithelial-mesenchymal transition (EMT) in RMS xenograft tumors **(A & B)** Immunofluorescence staining of E-cadherin (E-cad), Fibronectin, Snail and Twist following GANT-61 treatment in RD (A) and RH30 (B) cells-derived xenograft tumors as compared to their respective vehicle-treated control tumors. **(C)** Western Blot analysis showing expression of E-cad, N-cadherin (N-cad) and Twist in GANT-61 and-vehicle-treated tumors. Histograms represent dentiometric analysis of western blot bands and expressed as fold change. Photographs were captured at 40X magnification using Olympus BX51 with Olympus DP71 digital camera. Inset represents magnified area of the photomicrographs of 40X images. *P* value represents level of significant difference in GANT-61-treated and vehicle-treated controls.

Earlier, we have shown the combined role of Shh and Akt-mTOR signaling pathway in EMT regulation [25]. These signaling pathways are also known to regulate EMT in various cancer-types [36, 37]. Since we observed here that GANT-61 affects the expression of proteins in both of these pathways, we tested whether GANT-61 treatment could modulate the expression of proteins that regulate EMT. Immunofluorescence analysis showed that expression of the epithelial biomarker, E-cadherin was increased whereas mesenchymal biomarkers such as Fibronectin and Snail were decreased following GANT-61 treatment in both RD and RH30 cells-derived xenograft tumors as shown in Fig 5A & 5B. The transcription factors Twist and Slug, which are known EMT regulators, were reduced in GANT-61-treated tumors (Fig. 5A & 5B). Similar conclusion could be drawn based on western blot analysis. Data shown as histogram represents the dentiometric analysis of western blot (Fig. 5C).

### GANT-61-mediated RMS cell viability is reduced when combined with chemotherapeutic agents

**Figure 6:**
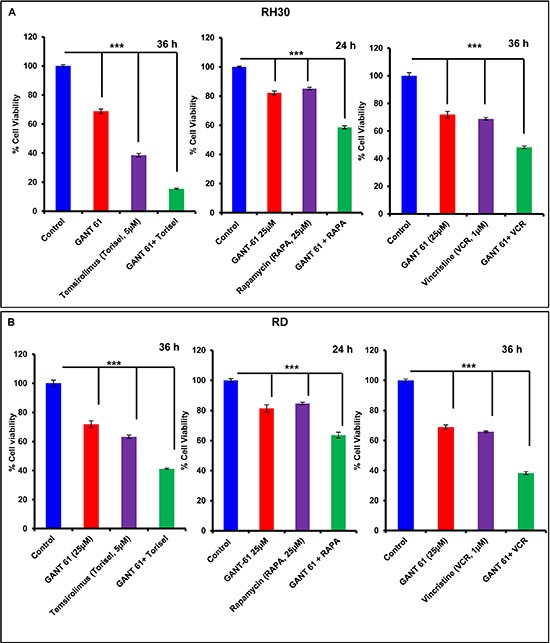
Combinatorial effects of chemotherapeutic drugs and GANT-61 on RMS cells MTT assay analysis of RH30 **(A)** and RD **(B)** cells showing changes in percent cell viability. Data are expressed as percentage of vehicle-treated controls taking as 100%. ***P > 0.001 value represents significant difference in various treatment groups. Human RMS cells were treated with either GANT-61 (25μM, 24 & 36 h) alone or in combination with temsirolimus (5μM, 36 h), rapamycin (25μM, 24 h) or vincristine (1μM, 36 h).

Next, we examined whether blocking GLI1/2 by GANT-61 sensitizes RMS cells to mTOR inhibitors and effectively diminish their survival. These studies are of potential importance for developing novel combinatorial approaches for killing refractory RMS and also for the dose reduction of highly toxic chemotherapeutics agents which are not well-tolerated in certain individual [38]. Temsirolimus and rapamycin are known mTOR inhibitors and vincristine is a mitotic inhibitor. These agents are included in the chemotherapeutic regimens for the treatment of RMS [38, 39]. Several recent ongoing clinical trials with these drugs in patients with recurrent and metastatic malignancy are in place [40]. Here, we demonstrated that treatment of RMS cells with temsirolimus and rapamycin in combination with GANT-61 significantly (***P value > 0.001) reduced the survival of both RH30 and RD cells as compared to single arm treatments (Fig. 6 A & 6B). Vincristine, another important chemotherapeutic drug frequently used for the treatment of RMS showed similar results against both RMS sub-types when compared with GANT-61 alone single arm treatment (Fig 6A & 6B). In these studies, we observed frequent alterations in cell morphology which was followed by cell death (Supplementary Fig. S4).

## DISCUSSION

At the time of diagnosis, RMS is clinically characterized as intermediate and high risk in 68% of cases and being metastatic in 16% cases [41]. A poor prognosis is known to be associated with fusion gene-negative aRMS and eRMS showing activation of the Hh signaling pathway [34]. Since, GLIs are the effector molecules of Shh signaling pathway, we utilized various cell lines derived from RMS patients to investigate whether expression of Gli1 mRNA or protein or both are enhanced. The high expression of GLIs may also have severe consequences not only in terms of RMS pathogenesis but also in attaining resistance to certain therapeutic modalities. Among the cell lines utilized in this study, RD was derived from the pelvic RMS developed in 7 year old female child who was treated with cyclophosphamide and radiation and found to have refractory diseases. The histologic appearances of tumor biopsies conferred an eRMS [42]. RH30, the other cell line in this study was derived from the bone marrow of a 16 year old male with untreated metastatic aRMS [42]. The observations in this study that enhanced expression of Gli1 and/or Gli2 occurs in these RMS cells provide a tempting scenario to investigate the role of Gli1/2 as molecular target for blocking pathogenesis of these deadly children's cancers since it is known that high expression of GLIs may also have severe consequences in attaining resistance to certain therapeutic modalities. After both *in vitro* and *in vivo* confirmation that RD and RH30 cells carry elevated Gli1 and Gli2 mRNA expression, we employed them to test the hypothesis that blocking Glis may provide an advantage in reducing growth of these tumors and that the residual tumors in the drug-treatment group might manifest a less invasive phenotype. This hypothesis was based on the fact that Hh signaling is known to be involved in augmenting EMT [25, 43, 44] and facilitates the development of an aggressive and invasive tumor phenotype in certain tumor models [36, 45]. Consistent with earlier published observations [18], we found that both RD and RH30 cells form stable xenograft tumors when subcutaneously injected to nude mouse. Indeed, our results demonstrated that GANT-61 dampens the proliferation signals in xenograft tumors derived from both of these cell lines. Its prolonged treatment reduces tumor growth in the GLI-inhibition-dependent manner. Although, earlier reports show that GANT-61 inhibits the growth of eRMS both *in vitro* and in xenograft model [9, 23] but these studies are largely descriptive and failed to provide a clear mechanistic basis for implicating GLIs as potent therapeutic target for RMS in general. Thus, particularly its effects remain largely undefined for aRMS. Recently Agyeman et al. demonstrated that by binding to the 5-zinc finger GLI1 protein at sites E119 and E167 in zinc fingers 2 and 3 GANT-61 inhibits GLI1 [46]. However, it is not clear whether similar mechanism underlie GLI2 inhibition. Results in this study demonstrated that the mechanism underlying inhibition in proliferation of both of these tumor cells is largely dependent on the ability of GANT-61 to slow down the cell cycle progression by arresting both aRMS and eRMS in G0/G1 phase. We also observed that this cell cycle arrest could be related to the GANT-61-mediated reduction in the expression of cyclin Ds which are known driver of the cell cycle from G1 to S phase. The additional observations in this study that GANT-61 mediates induction of cell cycle inhibitory protein p21 which binds to cyclin Ds/CDks and cyclin E/cdk2 protein kinase complexes to block their kinase activity provides strength to the notion that GANT-61 targets cell cycle regulatory proteins assembly. These data demonstrate that consequences of targeting GLIs on the growth inhibition of both major RMS sub-types could be mediated via slowing down the cell cycle progression.

Since, we found in this study almost equal effectiveness of GANT-61 against these two RMS sub-types, we conducted an additional experiment to assess whether GLIs may alter the growth of rhabdoid tumors as well. Rhabdoid cancer is rare lethal early childhood cancer [47]. A2O4 which earlier classified as RMS cells later showed to carry a SMARCB1 (SNF5) mutation and possess other characteristics of rhabdoid neoplasm [42, 48]. In this regard, we utilized A204 cells-derived xenograft tumor as model of rhabdoid tumors [21] and treated them with forskolin. Forskolin, is a known inducer of PKA, a kinase which by phosphorylating GLIs and promotes their degradation [49]. The reduced tumor growth associated with the inhibition of GLI by forskolin (Supplementary Fig. S5) suggests that GLIs are important therapeutic target for a wide variety of RMS sub-types irrespective of their genotype or differentiation states.

Another important finding in this manuscript is that GANT-61 by inhibiting GLIs alters the tumor phenotype which is reflected by the reduction in the expression of mesenchymal marker proteins with the concomitant enhancement in epithelial polarity marker protein. These data suggest that GANT-61 by inhibiting Shh signaling effector transcription factor could reduce EMT and consequently RMS tumor cells become less aggressive and invasive. These results are also consistent with the remarkable reduction in the proliferative potential of these cells as assessed by colony formation assay. Interestingly, forskolin-mediated modulation of GLI expression in rhabdoid A204 xenograft tumors was also associated with the reduction in EMT-related proteins further suggesting that the expression of GLIs may be associated with aggressive and invasive tumor phenotype.

The molecular mechanism of EMT modulating effects of GANT-61 seems to be mediated via down-regulation of AKT/mTOR signaling pathways. The dramatic decrease in p-AKT and p-P70S6k levels in GANT-61-treated RMS cells and tumors as observed here might play an important role in altering the metabolism-dependent phenotype of these tumors. The mechanism by which GANT-61 alters AKT/mTOR signaling pathway is not clearly understood at this time. Further in-depth studies are required to embark on the role of these pathways in abolishing drug resistance in recurrent and refractory RMS. However, the crosstalk between Shh and mTOR pathways is known to exist in other cancers [35]. Interestingly, earlier we also showed that treatment with rapamycin of A204 cells-derived xenograft tumors not only reduced mTOR signaling pathway but also reduced expression of GLIs suggesting an existence of possible crosstalk between the two pathways [21]. Our data that treatment of RD and RH30 cells with chemotherapeutic drug vincristine and mTOR inhibitors temsirolimus and rapamycin augments the ability of GANT-61 for enhanced killing of these tumor cells supports this notion.

In summary, these data provide evidence that targeting GLIs in various RMS sub-types may be successful in the management of relapsed and refractory RMS. These data also provide some evidence that GLIs play a role in developing an aggressive and invasive tumor phenotype. In this regard, a nexus between GLIs and AKT/mTOR might play a critical role. The remarkable efficacy of GANT-61 in combination with other chemotherapeutic drugs also point to the utility of this molecular target in sensitizing refractory tumors. Based on these data, we speculate that GLIs may provide important therapeutic target to develop combinatorial approaches for eliminating resistant and recurrent RMS by certain otherwise less effective therapeutic agents.

## MATERIALS AND METHODS

### Cell culture and reagents

Normal human smooth muscle (HA-VSMC), RMS (RH30, aRMS), (RD, eRMS) and rhabdoid cell line (A204) cells were procured from American Type Culture Collection (ATCC). While (CW9019, aRMS) and (SMS-CTR, eRMS) cells were gifted to JGP by Dr. Frederic G. Barr, Deputy Branch Chief and Senior Investigator Laboratory of Pathology, National Cancer Institute, USA. RMS cells were cultured in DMEM supplemented with 10% fetal bovine serum, 100 U/ml of penicillin, and 100 μg/ml of streptomycin at 37°C in a humidified atmosphere of 5% CO_2_. HA-VSMC were grown in F-12K medium supplemented with 0.05 mg/ml ascorbic acid, 0.01 mg/ml insulin, 0.01 mg/ml transferrin, 10 ng/ml sodium selenite, 0.03 mg/ml endothelial cell growth supplement (ECGS); fetal bovine serum to a final concentration of 10%, HEPES to a final concentration of 10 mM, TES to a final concentration of 10 mM. GANT-61 was purchased from Cayman (MI, USA). Rapamycin was purchased from Fujian Kerui Pharmaceutical Co., Ltd (Fuzhou, Fujian, China). Temsirolimus and vincristine were obtained from Pfizer (NY, USA) and Hospira (IL, USA) respectively. Primers used in this study were obtained from Invitrogen (Supplementary Table S1-I and II). Primary antibodies were purchased as described in supplementary Table S2. Various treatments of these cells were performed when their confluence reached to about 70-80%.

### Tumor xenograft study

Female mice (athymic nu/nu, 3-5 weeks, 25-30g) were purchased from NCI-Frederick Animal Production Program (Frederick, MD, USA). All animal procedures were performed according to guidelines and approvals of the Institute Animal Care and Use Committee of the University of Alabama at Birmingham (IACUC). For this study, RD and RH30 cells were detached by trypsinization, washed, and re-suspended in cold PBS. Animals were administrated 2.5 × 10^6^ RD or RH30 cells/200 μL PBS subcutaneously in each rear flank after that animals receiving each cell-type were divided into two groups (n = 5, each group). This experiment was continued until tumor size in control group exceeded 1cm^2^. At this time, both the vehicle-treated and the GANT61-treated groups were euthanized. GANT-61 stock solution was made in 100% ethanol and diluted in PBS. Tumors were measured by digital calibers and tumor volumes calculated using the formula volume = length × width × height plotted as a function of days on test. The subcutaneous tumor was removed and divided into two pieces, one of which was snap frozen in liquid nitrogen, and the other was fixed in 10% buffered formalin and embedded in paraffin.

### Hematoxylin and eosin (H&E)

Hematoxylin and eosin staining was performed exactly as described in our previously published study [50]. Briefly, tumors were fixed in 10% buffered formalin and embedded in paraffin. Tumor tissues were cut into sections (5 μm) using microtome (HM 325, Thermo scientific, MA, USA). Sections were deparaffinized in xylene and rehydrated. Tumor sections from each group were stained with H&E and examined for tumor histology under various magnifications using Olympus BX51 with Olympus DP71 digital camera.

### Immunohistochemical (IHC)/Immunofluorescence (IF) staining

For immunohistochemical staining, sections were de-paraffinized following rehydration and then incubated in antigen unmasking solution according to the manufacturer's instructions (Vector laboratories, Burlingame, CA, USA). To avoid nonspecific binding of antibodies, a blocking buffer of 2% BSA in PBS for 30 min at 37°C was used and then these sections were incubated with primary antibodies followed by a universal peroxidase-coupled secondary antibody and visualized with DAB substrate. For immunofluorescence staining, tissue sections were incubated with fluorescein-conjugated secondary antibodies for 1 h at room temperature and rinsed with PBS. Sections were mounted with DAPI and visualized under 40X magnification using Olympus BX51 with Olympus DP71 digital camera.

### RNA isolation and reverse transcriptase PCR

RNA isolation and reverse transcriptase PCR analysis was performed as described previously [51]. Briefly, total RNA was extracted from cells using TRIzol method (Invitrogen, CA, USA). mRNA (1 μg) was reverse-transcribed into cDNA by iScript cDNA synthesis kit (Bio-Rad, CA, USA). PCR products were run on 1.5% agarose gels and the relative quantification was done using Gel Documentation System (Bio-Rad laboratories, Segrate, Italy). GAPDH was used as an internal control. Primers employed in this study are listed in Supplementary Table S1-I.

### Quantitative real time PCR (qRT-PCR)

qRT-PCR analysis was performed using either SYBR green (Bio-Rad, CA, USA) fluorescent dye or TaqMan (Applied Biosystem, CA, USA) PCR master mix [51]. Total cDNA (250 ng) was used in a 20 μl reaction mixture with sequence specific primers. qRT-PCR reactions were carried out in triplicate using 7500 fast Real-Time PCR system (Applied Biosystem, CA, USA). Cycling conditions were 20s at 95° C followed by 40 cycles at 95°C for 3s and 60°C for 30s. Relative quantification of the steady state target mRNA levels was done after normalization of total amount of cDNA to GAPDH internal reference. Real time PCR primers employed in this study are listed in Supplementary Table S1-II.

### Protein quantification and Western blot (WB) analysis

Protein quantification and western blot analysis were performed as described earlier [50]. Protein lysate from various treatment groups were prepared using an ice cold lysis buffer (50mmol/L Tris, PH 7.5 Triton x-100, 0.25% NaF, 10mmol/L β- glycerolphosphate, 2mmol/L EDTA, 5mmol/L sodium pyrophosphate, 1mmol/L Na3VO4, 10mmol/L DTT and protease inhibitor) followed by centrifugation at 5000 rpm for 10 minute to obtain a clear supernatant. Protein assay was done using a DC kit (Bio-Rad, CA, USA). Lysate were mixed with 5X sample buffer (312.5 mM Tris-HCL, PH-6.8, 5%β-mercaptoethenol, 10% SDS, 0.5% bromophenol blue, 50% glycerol), boiled for 5 minute at 95°C and subjected to SDS-PAGE. Proteins were electrophoretically transferred to polyvinylidene difluoride membrane and then nonspecific site were blocked with 5% nonfat dry milk in Tris buffer saline tween-20 (TBST) (25 mmol/L Tris-HCL, PH-7.5, 150 mmol/L NaCl, 0.05% Tween-20) for 1 h at room temperature followed by probing with primary antibody overnight at 4°C or 2 h at room temperature. After washing, the membranes were incubated for 1.5 h with respective (anti-mouse, anti-rabbit or anti-goat immunoglobulin) HRP conjugated secondary antibody. The blots were developed with enhanced chemiluminescence (ECL) according to manufacturer's instructions (Amersham Bioscience).

### MTT assay

Cell viability was assessed by 3-(4,5-dimethylthiazol-2-yl)-2-5-diphenyl tetrazolium bromide (MTT) assay as described earlier [50]. Briefly, 10,000 cells per well were seeded into 96-well micro-culture plates at 37°C with 5% CO_2_ and allowed to attach for 24 h. Cells were treated with designated doses of GANT-61 and rapamycin for 24 h and incubated with MTT at final concentration 0.5 mg/ml for 4 h before the completion of exposure time at 37°C. Formation of MTT to formazon crystals by viable cells was assessed using 200 μL/well DMSO at room temperature for 15 min. Optical density was measured at 490 nm using micro-plate reader model 680 (Bio-Rad, CA, USA). The reduction in viability of cells in each well was expressed as the percentage of control cells.

### Morphological alterations

Morphological changes were assessed in cultured cells treated with various treatment groups. Phenotypic alterations such as cell roundness, shrinkage, blebbing etc. were recorded by the phase contrast microscopy (Olympus1X-S8F2, Japan).

### Cell cycle analysis

Flow cytometry analyses of the cell cycle were performed at various time intervals following treatment of RMS cells with GANT-61 at different concentrations. Briefly, cells were detached from plates with the help of Trypsin/EDTA, washed in phosphate-buffered saline, and fixed with chilled methanol for 4 h. Methanol-fixed cells were washed with PBS and incubated with RnaseA (Sigma) at 37°C in water bath for 1 h. Then, these cells were paletted out and re-suspended in DNA staining solution, propidium iodide (10 μg/ml) for 1 h and passed through BD FACS Calibur (BD biosciences, CA, USA). Samples were analyzed for cell cycle analysis.

### Clonogenic survival assay

RMS cells were seeded into 6-well plates at very low density of 800-1000 cells per well and were allowed to grow overnight. Then the plate attached cells were treated with GANT-61 at various concentrations or vehicle for 24 h. Drug or vehicle-treatment medium was replaced by fresh drug-free medium and cells were incubated for additional 10 days in humidified chamber at 37°C with 5% CO_2._ Cell colonies were fixed with cold methanol, stained with crystal violet, washed and air-dried. Blue colonies were scored and photographed. Each condition was replicated in triplicate. Data expressed as percent colonies formed relative to the control using the formula: (Average treated count)/(average control count) ×100.

### Statistical analysis

Results are expressed as mean ± standard error of mean (SEM). Statistical analysis between two groups was performed using Student's t test. P value of at least 0.05 represents the significant level from their respective groups.

## SUPPLEMENTARY FIGURES AND TABLES


