# Supplementary material for: GLI inhibitor GANT-61 diminishes embryonal and alveolar rhabdomyosarcoma growth by inhibiting Shh/AKT-mTOR axis
Source: Oncotarget. 2014 Oct 31;5(23):12151–65. doi: 10.18632/oncotarget.2569 (PMC4322980; doi:10.18632/oncotarget.2569)
Supplement: Supplementary file 1 [file oncotarget-05-12151-s001.pdf]

SUPPLEMENTARY FIGURES AND TABLES

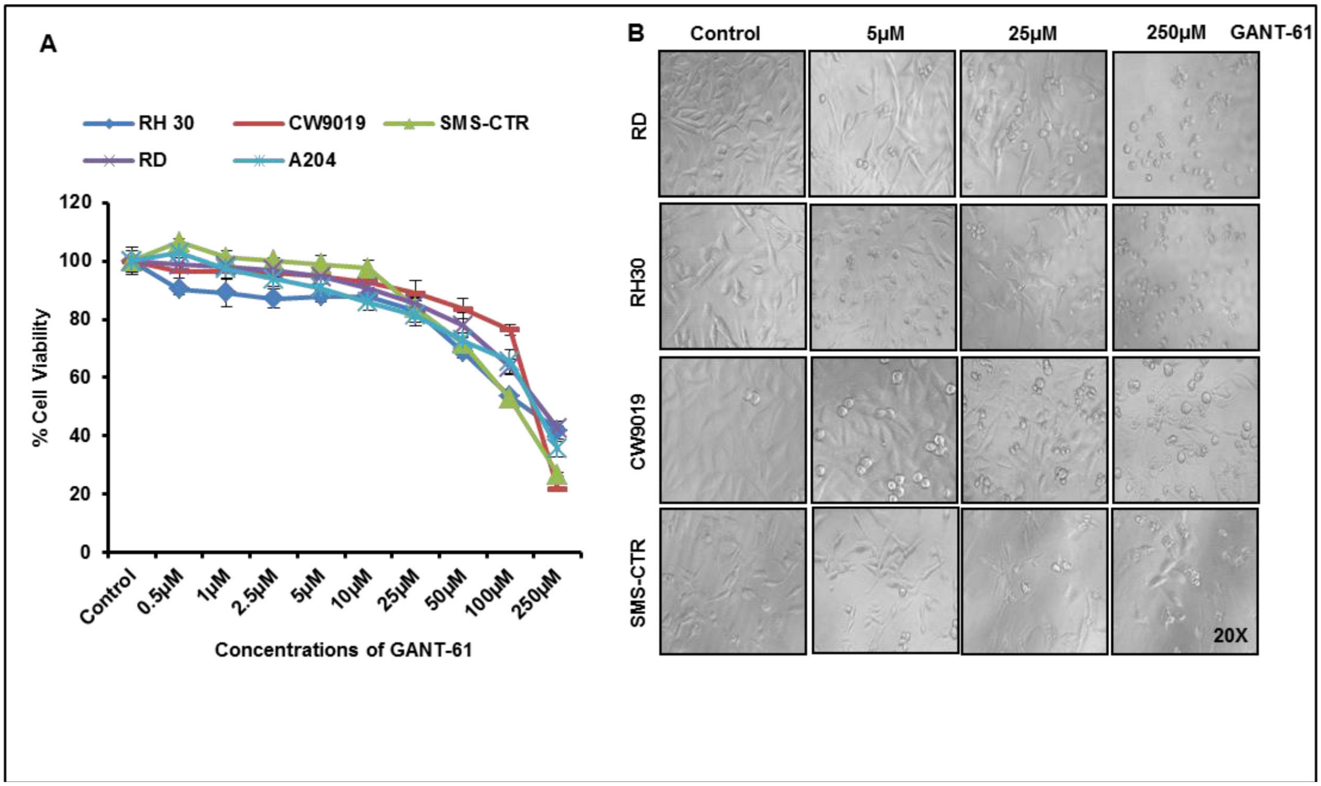

Supplementary Figure S1: Effects of GANT-61 treatment on cell viability. **(A)** MTT assay showing % cell viability of RMS cells treated with GANT-61 at various concentrations (0.5–250 µM) for 24 h. **(B)** Phase contrast microphotographs (20X) of RMS cells captured following GANT-61 treatment at concentrations range 5–250 µM for 24 h. GANT-61-treated RMS cells exhibited cell rounding, contraction of cytoplasmic membrane and blebbing.

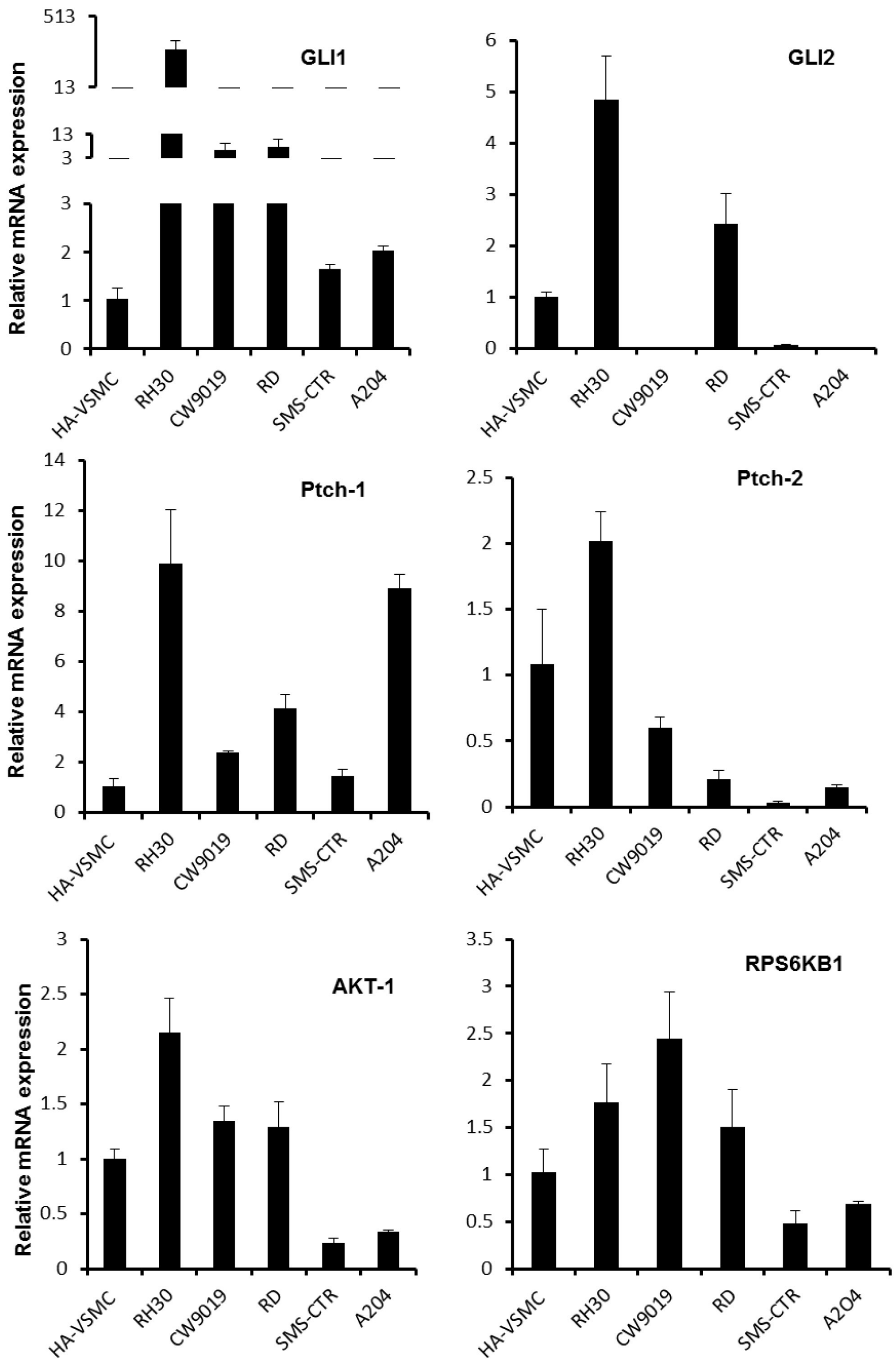

Supplementary Figure S2: Expression of Shh and Akt/mTOR pathway genes by Real Time PCR. mRNA expression analysis of Shh (Gli1, Gli2, ptch1 and ptch2) and Akt (Akt-1, RPS6KB1) signaling pathways related genes in aRMS (RH30 and CW0919), eRMS (RD and SMS-CTR) and rhabdoid A204 cells in comparison to human normal skeletal muscle (HA-VSMC). GAPDH is used as an endogenous control. Data are expressed as fold changes taking average of three different samples.

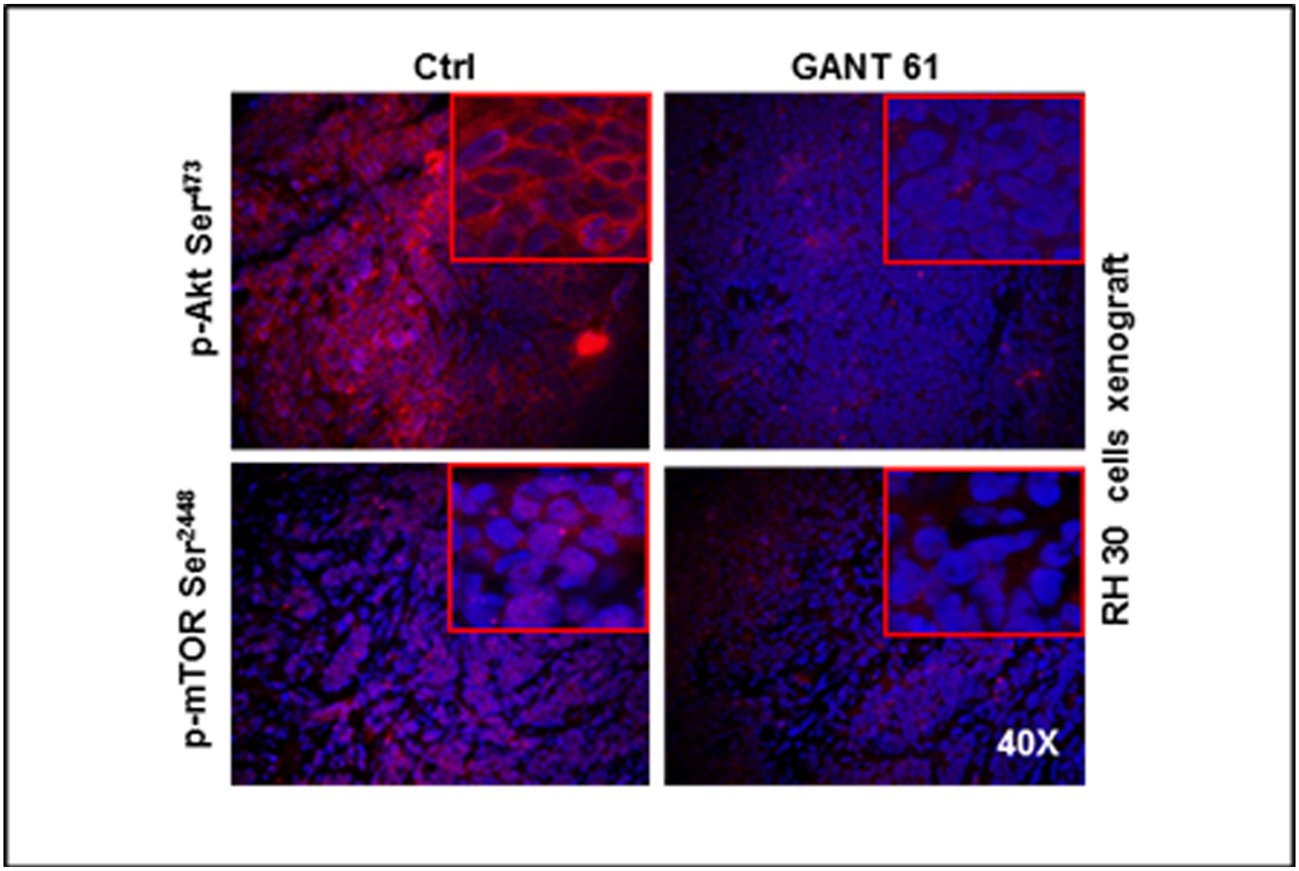

Supplementary Figure S3: Expression of p-AKT and p-mTOR in RH30 cells-derived xenograft tumors. Immunofluorescence staining of p-AKT Ser^{473} and p-mTOR Ser^{2448} from the 5 µM sections of GANT-61-treated RH30 cells-derived formalin fixed tumors vs. vehicle-treated tumors. Microphotographs were captured using Olympus1X-S8F2, Japan. Insets represent magnified area of the images.

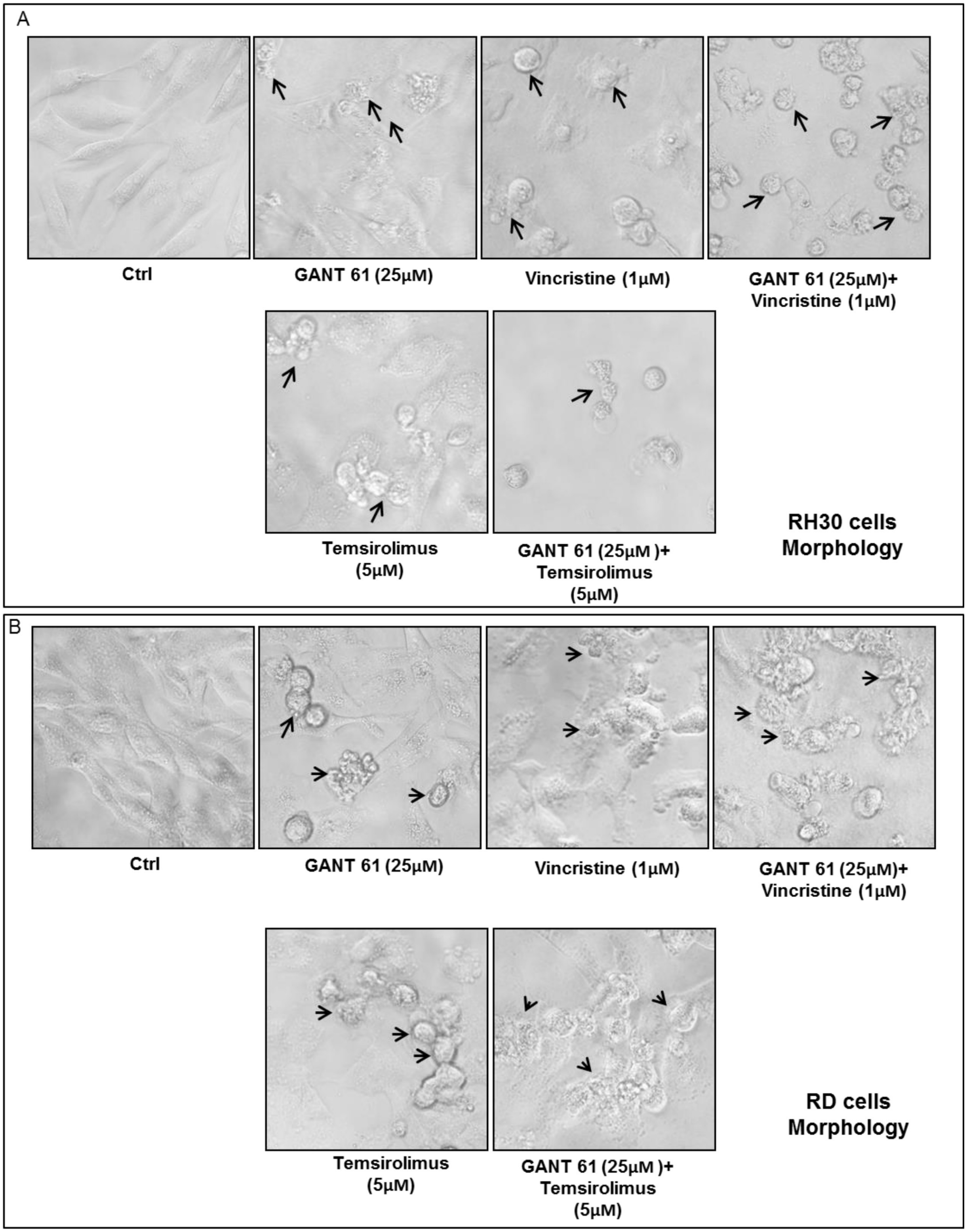

Supplementary Figure S4: Morphological alteration showing effects of temsirolimus and vincristine on cell death in GANT-61-treated RH30 and RD cells. Phase contrast microphotographs (40X) of RH30 (A) and RD (B) RMS cells captured following GANT-61 (25 µM) treatment either alone or in combination with Vincristine (1 µM) or Temsirolimus (5 µM) at 24 h. Arrows indicating the cell rounding and cytoplasmic contraction followed by cell death.

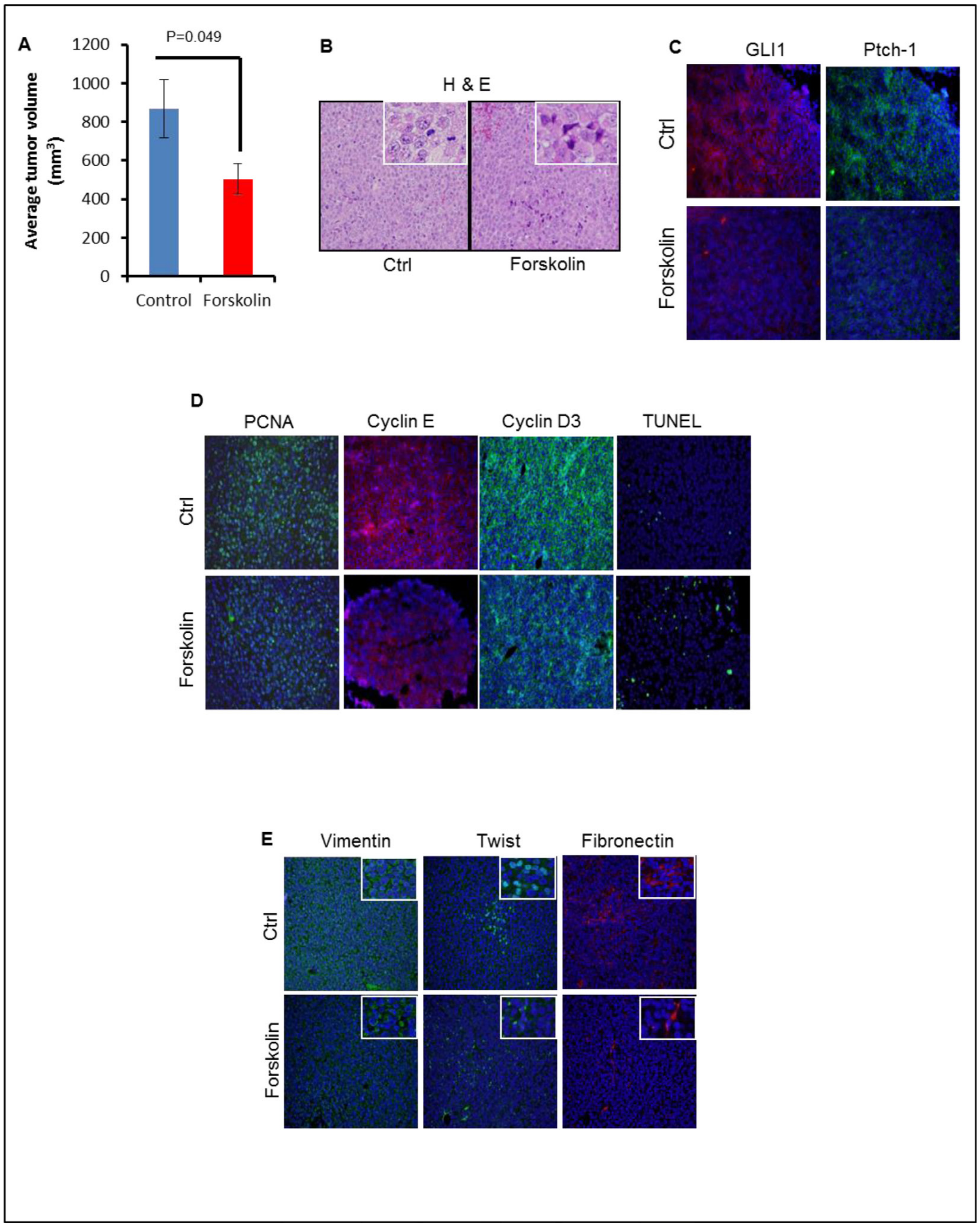

Supplementary Figure S5: Effects of forskolin on GLI expression in A204 cells-derived poorly differentiated xenograft rhabdoid tumor. **(A)** Graph representing treatment of nude mice bearing A204 cells derived xenograft tumors with forskolin. 5 nude mice each in vehicle and forskolin-treated groups were injected with $2X10^6$ A2O4 cells subcutaneously in each rear flank. Forskolin dose: 125µg/mouse (50mg/vial dissolved in 620µl Cremophor EL and 329µl 100% ethanol →50µg/µl stock, store at RT, in dark. It was diluted freshly in PBS by 1:80 and injected intra-peritoneally into mice daily. The control group animals were injected with vehicle. *P* value represents the significant difference when compared to vehicle-treated controls ($P = 0.049$). **(B)** Histology (H&E staining) of A204 tumors. Frequent mitosis in tumors from the control group could be noted while increased apoptosis in forskolin-treated group was apparent. **(C)** Immunofluorescence staining of tumor sections showing the expression of GLI1 and Ptch-1. Note a remarkable decrease in the expression of these proteins in forskolin treatment groups. **(D)** Immunofluorescence staining of tumor sections showing that the expression of proliferation biomarkers, PCNA, Cyclins E and D3. Apoptosis is represented by green TUNEL positive cells. **(E)** Immunofluorescence staining showing the expression of mesenchymal biomarkers, Vimentin, Twist and Fibronectin. Forskolin-treated tumors show reduced expression of these proteins. Microscopic photographs (20X) captured using Olympus1X-S8F2, Japan. Insets represent magnified area of the images.

Supplementary Table S1-I. List of reverse transcriptase PCR primers used in the study.

Primers	Sequences
Cyclin D1	F 5′-CTGGCGATGAACTACCTGGA-3′
	R 5′-GTCACACTTGATCACTCTCG-3′
Cyclin D2	F 5′-TTACCTGGACCGTTTCTTGG-3′
	R 5′-ATCCACGTCTGTGTTGGTGA-3′
Cyclin D3	F 5′-GTCTGTTCCCCCTTCACAAA-3′
	R 5′-AGCTGAGCAGAAAGCAAAGC-3′
Cyclin E	F 5′-CCATCCTTCTCCACCAAAGA-3′
	R 5′-AGCACCTTCCATAGCAGCAT-3′
GLI-1	F 5′-GACGGTTATCCGCACCTCAC-3′
	R 5′-AGGCTCACGCTTCTCCTCTC-3′
GLI-2	F 5′-CTCACCTCCATCAATGCCACGCCCA-3′
	R 5′-CCACCAGCATGTACTGCGCCTTGA-3′
GAPDH	F 5′-GGGGCTGGCATTGCCCTCAA-3′
	R 5′-GGCAGGGACTCCCCAGCAGT-3′

Supplementary Table S1-II. List of real time PCR primers used in the study.

Real time PCR primers	Cat No.	Company
Cyclin D1	Hs00765553-m1	Life technology
Cyclin D2	Hs00153380_m1	Life technology
Cyclin E1	Hs01026336-m1	Life technology
GLI-1	Hs01110766-m1	Life technology
AKT-1	Hs00178289-m1	Life technology
RPS6KB1 (pS6)	Hs00177357-m1	Life technology
GAPDH	Hs02758991-g1	Life technology

Supplementary Table S2. List of primary antibodies used in this study.

Antibody	Company	Application
PCNA	Santa Cruz	IHC/IF
Cyclin D1	Cell signaling	Western Blot/IF
Cyclin D3	Cell signaling	IF
Cyclin E	Santa Cruz	IF
Cleaved Caspase-3	Cell signaling	Western Blot
P^{21}	Cell signaling	Western Blot
GLI-1	Santa Cruz	IF
p-Akt (Thr^{308})	Cell signaling	Western Blot
p-Akt (Ser^{473})	Cell signaling	Western Blot/IF
Akt 1/2/3	Cell signaling	Western Blot
p-mTOR Ser^{2448}	Cell signaling	Western Blot /IF
p-P70S6K	Cell signaling	Western Blot
E-cadherin	Santa Cruz	IF
Twist	Santa Cruz	Western Blot/IF
Snail	Santa Cruz	Western Blot/IF
Fibronectin	Santa Cruz	IF
Vimentin	Santa Cruz	IF
β-actin	Sigma	Western Blot
